# Viral Hepatitis B/C Co-Infection and Its Association With Haematological and Virological Parameters in HIV Patients in Northern Ghana

**DOI:** 10.1155/anem/5212533

**Published:** 2025-06-30

**Authors:** Uzzah Mohammed Forgor, George Doopaar Billak, Nsoh Godwin Anabire, Gideon Kofi Helegbe

**Affiliations:** ^1^Department of Biochemistry and Molecular Medicine, School of Medicine, University for Development Studies, Tamale, Ghana; ^2^Department of Chemistry, University of Louisville, Louisville, Kentucky, USA; ^3^West African Centre for Cell Biology of Infectious Pathogens, Department of Biochemistry, Cell and Molecular Biology, University of Ghana, Accra, Ghana

**Keywords:** antiretroviral therapy, coinfection, haematological parameters, hepatitis B, hepatitis C, HIV, viral load

## Abstract

Human immunodeficiency virus (HIV) and viral hepatitis B (HBV) and C (HCV) share common routes of transmission and increase the morbidity and mortality of infected patients. In developing countries, including Ghana, HBV/HCV diagnoses are not routinely performed for patients in HIV clinics. Thus, the haematological impacts of hepatitis B/C are not evaluated before the inception of antiretroviral therapy (ART). This was a hospital-based cross-sectional study that assessed the prevalence of HBV and HCV infections among 135 HIV-1 infected patients in an HIV clinic in the Tamale Metropolis of Ghana using rapid diagnostic test kits. Haematological parameters and HIV load were evaluated and compared between HIV monoinfected and HIV-HBV or HIV-HCV coinfected patients. HIV-HBV and HIV-HCV coinfection rates were 8.9% and 5.9%, respectively. One participant (0.7%) was triply infected with HIV-HBV-HCV. HIV viral load was comparable in the different disease groups (*p* > 0.5 for all comparisons). Neutrophils and lymphocyte counts were lower in HIV/HCV coinfected patients in contrast to HIV-monoinfected patients (*p* > 0.05 for all comparisons). Significantly lower total WBC counts in HIV/HCV coinfected patients (*p* = 0.002) as compared to HIV monoinfected patients were observed. Generally, the rates of haematological abnormalities (anaemia, leucopenia, lymphocytopenia, neutropenia and monocytopenia) were higher in coinfected cases than in monoinfected cases. In conclusion, patients at HIV clinics in the Tamale Metropolis of Ghana have a high rate of HBV/HCV coinfection, which can have a significant negative influence on haematological counts, particularly lymphocyte counts. This highlights the necessity of routine testing for HBV/HCV among HIV clinic patients to influence the choice of ART drugs prescribed.

## 1. Introduction

Hepatitis B virus (HBV), hepatitis C virus (HCV), and human immunodeficiency virus (HIV) infections are significant threats to global health and contribute significantly to global deaths associated with infectious diseases. At the end of 2023, there were reportedly 39.9 million HIV/AIDS patients worldwide [1]. In Ghana, over 34,000 new HIV infections were reported in the first nine months of 2024 [2]. As of the end of June 2023, 6072 persons were reported to be living with HIV in the Northern Region of Ghana [3]. The infection rate is alarming and, therefore, causes serious health concerns.

HIV weakens the immune systems, making infected persons susceptible to opportunistic infections and diseases. Since HIV and viral hepatitis share a common route of infection, individuals who are infected with HIV have an increased risk of being infected with viral hepatitis [4]. The most common hepatitis viruses, hepatitis B and C, are a health threat because they kill 1.34 million people per year worldwide, almost as many as are killed by HIV [5]. Hepatitis B and C infections are common among people living with HIV (PLWH). Globally, the prevalence of HBV and HCV among PLWH is 7.4% [1] and 2.4%, respectively [6]. Evidence indicates that coinfections with HIV, HBV, and HCV are widespread in sub-Saharan Africa; the mean prevalence of coinfection with HIV and HBV is 15%, while the rate of coinfection with HIV and HCV is 7% among HIV-positive individuals. [7]. Depending on geographic factors, risk categories, and the type of exposure, different populations have varying prevalence rates of HIV, HBV, and/or HCV. [8]. In Ghana, the prevalence of HIV and HBV coinfection ranges from 11% to 15% [9], while the prevalence of HIV and HCV coinfection has been reported to be less than 6% from studies conducted in various parts of Ghana [10–12].

Hepatitis B/C and HIV coinfection have become the leading cause of morbidity and mortality among coinfected patients due to liver-related disease, even after the introduction of life-saving antiretroviral medications [13]. In the coinfection of hepatitis and HIV, the presence of one virus influences the natural history of the other virus. HIV increases the natural course of HBV and HCV and, therefore, increases the progression of liver-related complications [9]. The progression of liver-related complications to cirrhosis in HIV-positive patients is nearly three times faster as compared to a non-HIV-infected patient [14]. Consequently, HIV patients who have viral hepatitis B or C are more likely to develop chronic kidney and liver disease [4] and experience more clinical symptoms, such as the impaired immune response during antiretroviral therapy (ART) [15]. ART drugs have been linked to a variety of side effects, including hepatotoxicity [16] and haematological abnormalities [17, 18]. HIV patients on ART are reported to have haematologic abnormalities such as peripheral blood cytopenia, neutropenia, anaemia and thrombocytopaenia [18], with anaemia being the most common of the abnormalities, and associated with faster HIV disease progression and mortality [16]. Monitoring some haematological indices such as haemoglobin (Hb), packed cell volume, platelet count, and total and differential white cell count gives an important glance at HIV disease progression as well as provides a proper indication of the response to ART in resource-limited settings [19]; however, assessment of viraemia is a golden standard method to diagnose ART failure as well as the progression of the disease [20].

Before and during ART, HIV-infected patients must be screened for HCV and HBV infections, as the presence of these viruses in a patient may influence the medications that should be administered. [21]. Even though HBV and HCV are serious public health problems in Ghana, systematic testing for these diseases among HIV-positive people is nonexistent or steadily reducing in resource-limited settings, including the Northern Region of Ghana. Due to a lack of funding, testing supplies are few in treatment facilities, which has a detrimental effect on the effectiveness of the ART medications used to treat HIV-infected patients. Therefore, we conducted this study to ascertain the prevalence of HBV and HCV infection among HIV-infected patients in the Tamale Metropolis of Ghana as well as to ascertain its association with haematological and HIV virological parameters.

## 2. Methods

### 2.1. Study Design and Site

A hospital-based cross-sectional study was conducted in the sexually transmitted infections (STI) clinic of the Tamale Teaching Hospital (TTH) from December 2018 to March 2019. The TTH is the largest public hospital in the northern part of Ghana, and it is the third tertiary teaching hospital in Ghana. The TTH is a well-known area in Tamale, easily located from the city's centre. The geographical location of the hospital and the commercial nature of Tamale make the hospital easily accessible and the preferred referral hospital for large catchment areas in Northern and Southern Ghana as well as some neighbouring countries such as Burkina Faso, Togo and Cote d'Ivoire [21].

### 2.2. Sampling

A total of 135 newly diagnosed HIV-1-infected patients who were naïve to ART were recruited for the study during their first clinic visit. The recruitment process for subjects was performed during their routine clinic visit days, and only patients who agreed to the consent given to them were recruited. All HIV-1-infected patients were eligible for recruitment to this study. The exclusion criteria for this study were patients who were unwilling to take part in the research and individuals who were infected with HIV-2. A well-structured questionnaire was administered to each participant to collect their socio-demographics (including sex, age group, religion and highest level of education) and the likely cause of the infection.

### 2.3. Collection of Blood Samples and Laboratory Investigations

Venous blood samples (5 mL) were collected aseptically into K_3_ EDTA tubes and used for rapid diagnostic (serological) detection of HBV and HCV, full blood count, and quantification of viral (HIV-1 RNA) load. A portion (3 mL) of the blood was centrifuged at 3000 rpm for 5 min, and the plasma was taken for hepatitis B and C screening and viral (HIV-1 RNA) load quantification. Rapid diagnostic test kits were produced by Guangzhou Wondfo Biotech Company Limited, China. Participants were screened for HBV and HCV by detecting the presence of hepatitis B surface antigen (HBsAg) and antibodies to Hepatitis C (Anti-HCV). The procedures, results and interpretations for the HBV and HCV tests were carried out according to the manufacturer's protocol [22]. Briefly, three drops of serum were added to each test kit well. The set-up was allowed on sitting, and the result was read after 15 min as either positive or negative based on the presence or absence of HBsAg for HBV detection and anti-HCV antibodies for HCV detection. Those positive for HBsAg and HCV were not confirmed using PCR technology.

Cobas Ampliprep/Cobas Taqman Analyzer (USA) was used to measure HIV viral load. The procedure for HIV viral load determination was conducted by strictly following the manufacturer's instructions. [23]. On the whole blood samples taken, a full blood count was performed using the Urit 5250 haematology analyzer (China) and following the instructions prescribed by the manufacturer [24].

### 2.4. Haematological Parameters and Definitions

The complete blood count parameters assessed in this study include Hb, white blood cells (WBC), lymphocytes, neutrophils and monocytes. Haematological abnormalities (cytopenias) were assessed as well and include anaemia (Hb < 12 g/dL for nonpregnant females and Hb < 13 g/dL for males) [25], leucopenia (WBC < 4.0 × 10^9^ cell/L), lymphocytopenia (lymphocyte < 1 × 10^9^ cells/L), neutropenia (neutrophils < 1.5 × 10^9^ cell/L) and monocytopenia (< 0.2 × 10^9^ cell/L) [26].

### 2.5. Ethical Approval

The study was approved by the institutional review board of the Navrongo Health Research Centre (ethical approval ID: NHRCIRB342). An authorization was granted by the STI clinic to enable the recruitment of participants for the study. Written informed consent was provided by each study participant.

### 2.6. Statistical Analysis

Data were analysed using SPSS version 20. The hepatitis prevalence rate among HIV-positive patients was calculated from the total numbers that were serologically tested for both HBV and HCV. Continuous variables between the two groups were compared with a *t*-test and presented as mean ± standard deviation (SD). Categorical variables were presented as frequency (*n*) and percentages (%) and compared using Pearson's Chi-square test or Mann–Whitney *U* test. The HIV viral load was transformed into Log_10_ and represented as Log_10_VL ± Uncertainty. The significant level was 95% confidence, and a *p* value < 0.05 was considered statistically significant.

## 3. Results

### 3.1. Sociodemographic Information Among the Study Participants

Of HIV patients attending the STI clinic in the TTH during the study period, 135 patients consented and were recruited to take part in the study. The socio-demographic information of the participants is shown in Table 1. Among the study subjects, the majority fell above 40 years (43.0%) and were females (71.1%). Most were Muslims (72.6%) and lived in the urban area (80.0%), predominantly in the Tamale metropolitan area. Most of the participants were married (55.6%), uneducated (37.8%), involved in informal occupations (60.0%) and earned low income (65.9%).

### 3.2. Likely Cause of Infection

Most of the participants were not certain about the likely cause of their infection since 65.9% of the participants were unaware of the possible cause of the infection (Table 2). One respondent had a history of using sharp syringes. Unprotected sex and family history accounted to 26.7% and 6.7% of the likely cause of infection among the study participants (Table 2).

### 3.3. Seroprevalence of Viral Hepatitis B and C

Twenty-one (21) individuals, representing 15.6% of the 135 study participants, were coinfected with viral hepatitis (Table 3). The serological prevalence of HBsAg was higher, with a percentage of 8.9%, as compared to the seroprevalence of HCV (5.9%), which was identified by the presence of antibodies to HCV (Table 3). One participant was multiinfected with HBV and HCV. With regards to socio-economic determinants, the seroprevalence of hepatitis B/C was higher in females (66.7%) than in males (33.3%) (Table 3). The seroprevalence of hepatitis B/C was also higher in participants involved in informal occupation (66.7%), married individuals (66.7%) and those with lower levels of formal education (uneducated and basic education; 66.9%) (Table 3).

### 3.4. Virological and Haematological Parameters Across Disease Groups

Comparison analysis showed similar levels of HIV viral load in HIV monoinfected and HIV/HBV coinfected patients, in HIV monoinfected and HIV/HCV coinfected patients and HIV/HBV coinfected and HIV/HCV coinfected patients (*p* > 0.05 for all comparisons, Table 4). Comparison analysis also showed similar levels of haematological indices (Hb, WBC, neutrophils, lymphocytes and monocytes) between HIV monoinfected and HIV/HBV coinfected patients (*p* > 0.05 for all comparisons, Table 4). Similar levels of haematological indices were observed between HIV monoinfected and HIV/HCV coinfected patients (*p* > 0.05 for all comparisons, Table 4). However, WBC counts were significantly lower in HIV/HCV coinfected patients in contrast to HIV monoinfected patients (*p* < 0.05 for all comparisons, Table 4). All the haematological counts were also similar between HIV/HBV coinfected and HIV/HCV coinfected patients (*p* > 0.05, Table 4).

### 3.5. Prevalence of Haematological Abnormalities Among Participants

The prevalence of anaemia, leucopenia, lymphocytopenia, neutropenia and monocytopenia was compared across the disease groups. Generally, the cytopenias (anaemia; at least 39.0%, leucopenia; at least 51.0%, lymphocytopenia; at least 20.0%, neutropenia at least 40.0% and monocytopenia; at least 37.8%) were very prevalent among the disease groups (Table 5). A general trend observed was that the prevalence of the cytopenias was higher in coinfected cases relative to monoinfected cases, though no significant statistical differences were established (*χ*^2^ *<* 2.2; *p* > 0.3 for all comparisons, Table 5). Generally, the rates of cytopenias were higher in HIV/HCV coinfected cases than in HIV/HBV coinfected cases. However, no significant statistical differences were observed (*χ*^2^ *<* 1.3; *p* > 0.5 for all comparisons, Table 5). While we aimed to quantify the entire samples across the entire sample, we were able to obtain complete data for 73.33% (99/135) of the study population. The 36 samples that could not be quantified were due to limited resources and time to complete the study.

## 4. Discussion

### 4.1. Prevalence of Viral Hepatitis B/C

This study reports a combined prevalence of 15.6% for viral hepatitis B/C among HIV patients in the Tamale Metropolis of Ghana. The high rate of HBV infection (8.9%) among the study participants is comparable to the 8.8% prevalence reported in the Eastern region of Ghana [27], higher than rates reported in the Central region of Ghana (Cape Coast; 6.1% [10]) and the Volta region of Ghana (Ho; 7.0% [28]) but lower than rates ranging between 12.4%–24.4% reported in other parts of Ghana [4, 11, 12, 29–31]. Compared to other Sub-Saharan African countries, the rate of HIV/HBV coinfection in this study is lower than the 12.3% reported in the north-western part of Nigeria [32] and the 23.7% reported in Cameroon [33] but higher than the prevalence rate of 7.1% reported in Ethiopia [34]. The 5.9% rate of HIV/HCV coinfections detected in this current study is comparable to the rate of 5.5% reported in Kumasi, Ghana [11], higher than the rate of 0.5% in Cape Coast, Ghana [10], 1.5% in the Volta and Oti Regions of Ghana [28] and the rate of 3.6% in Accra, Ghana [12], but lower than the rate of 6.7% recorded by another study in Cape Coast, Ghana [31]. Compared to other African countries, the HIV/HCV rate in this study is lower than the rate of 7.2% reported in Cameroon [33] but higher than the rate of 1.6% in the north-western part of Nigeria [32]. Coinfection of viral hepatitis among HIV patients is known to vary from one geographic region to another, even within the same country [8]. Despite the trend being unclear, the heterogeneity in the rates of HIV/HBV and HIV/HCV coinfections across Ghana and other African countries may also be due to variations in how the viruses are acquired and transmitted among the various research populations. Although previously unreported in different regions of Ghana, the triple HIV/HBV/HCV infection prevalence rate of 0.7% compares with the incidence of 0.8% recorded in Ethiopia [34]. Compared to HIV/HBV and HIV/HCV coinfection, less information is available on triple coinfection of HBV, HCV, and HIV in Sub-Saharan Africa. The fact that HCV is less common in sub-Saharan Africa than in Europe [5], despite the region's high rates of HIV and HBV prevalence, may have a link to the dearth of data on triple HIV/HBV/HCV infections. More so, while HIV and HBV infect individuals mainly through sexual and perinatal transmissions in Africa [9], HCV is less efficiently transmitted through these routes [35]. As a result, the possibility of triple infection of these viruses is less in Africa, giving a clear explanation of the lower prevalence of HIV-HCV-HBV in the study population.

Our research relied upon rapid diagnostic kits to screen for HBV and HCV. It would have been an added strength to the study if the infections had been confirmed with a more specific technique, the PCR, but this was not performed.

### 4.2. Sociodemographic Determinants and Prevalence of Viral Hepatitis B/C

Higher rates of viral hepatitis B/C among female study participants than among male study participants may reflect the fact that women are more sensitive to changes in their health and are under pressure to seek and receive medical attention. In contrast, men may be forced to prove their manliness by ignoring symptoms of the infection to uphold the socio-culturally assigned role as the family's breadwinners [36, 37]. These claims may help to explain why more women than men are HIV-positive [38], as was also seen in this current study. Coinfection of viral hepatitis and HIV predominated among married people, indicating that transmission through sexual activities may be the most typical route of transmission of viral hepatitis and HIV in the Tamale Metropolis of Ghana. More so, most of the study participants were Muslims, and polygamy, as commonly practiced by Muslims, may be seen as a contributory factor. However, this may not be substantiated as it has been reported that polygamy is linked to the benign spread of HIV in Sub-Saharan Africa [39]. Due to the increased risk of infection from other transmission channels [40], this does not indicate that singles (divorced and unmarried) are at minimal risk of infection. The high rate of viral hepatitis and HIV coinfection among the uneducated and those with a low level of formal education is a sign of the lack of knowledge about the potential causes and modes of transmission of these infections. Due to their poor socioeconomic level, people who work in informal occupations may engage in behaviours and practices that contribute to the spread of HIV and hepatitis. Unfortunately, our study did not assess the risk factors that can predict viral hepatitis B/C coinfection among the study participants because the risk factors for viral hepatitis and HIV acquisition were not adequately recorded or accounted for by the participants. The study mainly discussed the socio-demographic determinants based on proportions, as captured in the presentation of the results. Among the study participants, the status of hepatitis B/C infection was unknown, which could have influenced the outcomes and interpretations of the results presented here.

### 4.3. Impact of Coinfection on HIV Viraemia

Untreated hepatitis coinfection reduces the benefits of successful HIV treatment by accelerating the advancement of hepatitis B and/or C-related liver disease and early death. After the start of ART, studies from various endemic countries have revealed that HBV/HIV and HIV/HCV coinfected patients show a slow rate of immunologic recovery and a poor virologic response [41, 42]. However, in this study, coinfections of HIV and viral hepatitis B/C did not cause any significant impact on the HIV viral loads in the different groups of the study population. This may be because the study participants were naïve to ART (newly confirmed cases). Additionally, the stages of the HBV and HCV infections were unknown, and it is possible that the infections had not advanced to a point where they could have affected HIV virologic responses. That notwithstanding, this observation corroborates with other findings in Ghana, other African countries and in Europe [11, 12, 43, 44].

### 4.4. Impact of Coinfection of Haematological Indices

Haematological abnormalities (such as anaemia, lymphocytopenia, leucopenia, neutropenia and monocytopenia [45–47]) are commonly observed in individuals infected with HIV, and the rates are observed to be higher, though not statistically significant, in those coinfected with HIV and viral hepatitis B/C, as observed in this study. The rates of haematological abnormalities in the HIV monoinfected cases are quite comparable and fall within the range of the rates reported in earlier studies in Ghana [45, 46] and other parts of Africa [47]. Increased rates of haematological abnormalities in coinfection cases can result from direct viral effects on the bone marrow, immune-mediated destruction of blood cells, or drug toxicity from ART and hepatitis B/C medications. With regards to impacts on absolute counts of haematological indices, the study found no statistically significant difference in the WBC count between HIV monoinfected and HIV/HBV coinfected patients. This finding is consistent with the study that found that HIV/HBV coinfection does not cause a significant change in the WBC count relative to HIV monoinfection [19]. However, the findings on the WBC count contradict the results of another study in Nigeria [48]. HIV/HCV coinfection impacted blood indices significantly, particularly the immunological parameters such as the WBC, neutrophils and lymphocytes among the study participants. HIV/HCV coinfection promotes lymphocyte destruction, which leads to a decrease in the CD4 count, which results in the decline in the total lymphocyte count in coinfected patients [49]. Moreover, HCV has been shown to infect and replicate in B cells, T cells and monocytes. [15]. These factors, as mentioned earlier, may have accounted for the significantly lower levels of lymphocytes and WBCs in HIV/HCV coinfected patients in this study.

## 5. Conclusion

This is the first-ever study reporting triple HIV/HBV/HCV infection in an individual in the study area and Ghana at large. Our findings demonstrate that patients at HIV clinics in the Tamale Metropolis of Ghana have a high rate of viral hepatitis B/C coinfection, which can have a major negative influence on haematological counts, particularly lymphocyte counts as seen in HIV-HCV coinfection cases. This highlights the necessity of routine testing for viral hepatitis B/C among HIV clinic patients to influence the choice of ART drugs prescribed. The findings also show that HIV patients in Tamale are unaware of the factors that lead to viral hepatitis and HIV infections, which may be related to the poor socioeconomic status of most study participants. This supports the need for actions to improve socioeconomic conditions, such as educating and raising knowledge of hepatitis acquisition and prevention among HIV patients to lessen coinfection of HBV and HCV.

Future research with a higher sample size and extending the duration and type of the study will help validate the results of this current investigation, especially the prevalence of triple HIV/HBV/HCV infection and the impact of coinfection on haematologic and virological parameters, given that the sample size employed in this study was slightly lower.

## Figures and Tables

**Table 1:** Sociodemographic characteristics of the study participants with HIV in Tamale, Ghana.

Characteristic	Category	Total (*N* = 135)	HIV mono (*n* = 114)	HIV and viral hepatitis coinfection (*n* = 21)
Age, years	< 20	6 (4.4)	6 (5.3)	0 (0)
	20–25	10 (7.4)	9 (7.9)	1 (4.8)
	26–30	19 (14.1)	15 (13.2)	4 (19.0)
	30–35	24 (17.8)	22 (19.3)	2 (9.50)
	36–40	18 (13.3)	13 (11.4)	5 (23.8)
	> 40	58 (43.0)	49 (42.9)	9 (42.9)

Sex	Female	97 (71.9)	83 (72.8)	14 (66.7)
	Male	38 (28.1)	31 (27.0)	7 (33.3)

Religion	Christian	37 (27.4)	30 (26.3)	7 (33.3)
	Muslim	98 (72.6)	84 (73.7)	14 (66.7)

Residence	Urban	108 (80.0)	92 (80.7)	16 (76.2)
	Rural	27 (20.0)	22 (19.3)	5 (23.8)

Occupation	Unemployed	35 (25.9)	31 (27.2)	4 (19.0)
	Informal	81 (60.0)	67 (58.8)	14 (66.7)
	Formal	19 (14.1)	16 (14.1)	3 (14.3)

Marital status	Divorced	22 (16.3)	17 (14.9)	5 (23.8)
	Married	75 (55.6)	61 (53.5)	14 (66.7)
	Single	22 (16.3)	22 (19.3)	0 (0.00)
	Widowed	16 (11.9)	14 (12.3)	2 (9.5)

Education	Basic	42 (31.1)	35 (30.7)	7 (33.3)
	Secondary	19 (14.1)	15 (13.2)	4 (19.0)
	Tertiary	23 (17.0)	20 (17.5)	3 (14.3)
	Uneducated	51 (37.8)	44 (38.6)	7 (33.3)

Economic status	High income	4 (3.0)	4 (3.5)	0 (0)
	Low income	89 (65.9)	73 (64.0)	15 (71.4)
	Middle income	42 (31.1)	36 (31.6)	6 (28.6)

**Table 2:** Likely cause of infection among the study participants with HIV in Tamale, Ghana.

Likely cause of infection	Frequency (*N* = 135)	Percentage (%)
Birth (family history)	9	6.7
Sharps/syringes/blades	1	0.7
Unknown	89	65.9
Unprotected sex	36	26.7

**Table 3:** Seroprevalence of hepatitis B and C among the study participants with HIV in Tamale, Ghana.

Parameter	Category	Frequency (n)	Percentage (%)
Serological marker	HBsAg	12	8.9
	Anti-HCV	8	5.9
	HBsAg + Anti-HCV	1	0.7

Sex	Female	14	66.7
	Male	7	33.3

Occupation	Unemployed	4	19.0
	Informal	14	66.7
	Formal	3	14.3

Marital status	Divorced	5	23.8
	Married	14	66.7
	Widowed	2	9.5

Educational status	Basic education	7	33.3
	Secondary education	4	19.0
	Tertiary education	3	14.3
	Uneducated	7	33.3

**Table 4:** Virological and haematological parameters of the study population.

Parameter	Disease groups		*p* value	Disease groups		*p* value	Disease groups		*p* value
	HIV monoinfection (*n* = 114)	HIV/HBV coinfection (*n* = 12)		HIV monoinfection (*n* = 114)	HIV/HCV coinfection (*n* = 8)		HIV/HBV coinfection (*n* = 12)	HIV/HCV coinfection (*n* = 8)	
	Log_10_ VL			Log_10_ VL			Log_10_ VL		
HIV viral load (copies/mL)	5.458 ± 0.117	5.175 ± 0.409	0.599	5.458 ± 0.117	4.944 ± 0.970	0.893	5.175 ± 0.409	4.944 ± 0.970	0.769

	**Mean ± SD**			**Mean ± SD**			**Mean ± SD**		

Haemoglobin (g/dL)	11.55 ± 1.85	11.73 ± 2.33	0.820	11.55 ± 1.85	10.75 ± 2.13	0.369	11.73 ± 2.33	10.75 ± 2.13	0.408
WBC (×10^9^ cells/L)	4.16 ± 2.22	3.64 ± 1.81	0.486	4.16 ± 2.22	3.07 ± 0.49	**0.002**	3.64 ± 1.812	3.07 ± 0.49	0.371
Neutrophils (×10^9^ cells/L)	1.6 ± 1.42	1.47 ± 0.81	0.784	1.6 ± 1.42	1.12 ± 0.745	0.201	1.47 ± 0.81	1.12 ± 0.745	0.395
Lymphocyte (×10^9^ cells/L)	1.72 ± 1.71	1.59 ± 0.75	0.812	1.72 ± 1.71	1.52 ± 0.96	0.649	1.59 ± 0.75	1.52 ± 0.96	0.875
Monocytes (×10^9^ cells/L)	0.28 ± 0.21	0.51 ± 0.68	0.316	0.28 ± 0.21	0.27 ± 0.24	0.720	0.51 ± 0.68	0.27 ± 0.24	0.326

*Note*: Comparisons were evaluated using a *t*-test. The *p* value is considered statistically significant at < 0.05 (2-tailed) and presented in bold in the table.

**Table 5:** Distribution of haematological abnormalities across the disease groups.

Haematological abnormalities		Disease groups		(*χ*^2^), *p* value	Disease groups		(*χ*^2^), *p* value	Disease groups		(*χ*^2^), *p* value
		HIV monoinfection (*n* = 82)	HIV/HBV coinfection (*n* = 10)		HIV monoinfection (*n* = 82)	HIV/HCV coinfection (*n* = 6)		HIV/HBV coinfection (*n* = 10)	HIV/HCV coinfection (*n* = 6)	
		*n* (%)			*n* (%)			*n* (%)		
Anaemia	Yes	32 (39.0)	5 (50.0)	(1.397) 0.497	32 (39.0)	3 (50.0)	(0.395) 0.821	5 (50.0)	3 (50.0)	(0.208) 0.901
	No	50 (61.0)	5 (50.0)		50 (61.0)	3 (50.0)		5 (50.0)	3 (50.0)	

Leucopenia	Yes	51 (62.2)	6 (60.0)	(0.737) 0.692	51 (62.2)	3 (50.0)	(0.399) 0.819	6 (60.0)	3 (50.0)	(0.357) 0.836
	No	31 (37.8)	4 (40.0)		31 (37.8)	3 (50.0)		4 (40.0)	3 (50.0)	

Lymphocytopenia	Yes	29 (35.4)	2 (20.0)	(1.940) 0.379	29 (35.4)	2 (33.3)	(0.720) 0.964	2 (20.0)	2 (33.3)	(0.556) 0.757
	No	53 (64.6)	8 (80.0)		53 (64.6)	4 (66.7)		8 (80.0)	4 (66.7)	

Neutropenia	Yes	48 (58.5)	4 (40.00)	(2.149) 0.342	48 (58.5)	4 (66.7)	(0.237) 0.888	4 (40.0)	4 (66.7)	(1.250) 0.535
	No	34 (41.5)	6 (60.0)		34 (41.5)	2 (33.3)		6 (60.0)	2 (33.3)	

Monocytopenia	Yes	31 (37.8)	4 (40.00)	(0.829) 0.661	31 (37.8)	2 (33.3)	(0.118) 0.943	4 (40.0)	2 (33.3)	(0.278) 0.870
	No	51 (62.2)	6 (60.00)		51 (62.2)	4 (66.7)		6 (60.0)	4 (66.7)	

*Note:χ* ^2^: Chi-square value, analysed using the Chi-square or Fisher's exact test. The *p* value is statistically significant at < 0.05 (2-tailed).

## Data Availability

The data for the study are available upon reasonable request.
